# Eliciting Big Data From Small, Young, or Non-standard Languages: 10 Experimental Challenges

**DOI:** 10.3389/fpsyg.2019.00313

**Published:** 2019-02-14

**Authors:** Evelina Leivada, Roberta D’Alessandro, Kleanthes K. Grohmann

**Affiliations:** ^1^Department of Language and Culture, UiT The Arctic University of Norway, Tromsø, Norway; ^2^Utrecht Institute of Linguistics, UiL-OTS, Utrecht University, Utrecht, Netherlands; ^3^Department of English Studies, University of Cyprus, Nicosia, Cyprus

**Keywords:** fieldwork, rich data, big data, experimental design, dialect, sign language

## Abstract

The aim of this work is to identify and analyze a set of challenges that are likely to be encountered when one embarks on fieldwork in linguistic communities that feature small, young, and/or non-standard languages with a goal to elicit big sets of rich data. For each challenge, we (i) explain its nature and implications, (ii) offer one or more examples of how it is manifested in actual linguistic communities, and (iii) where possible, offer recommendations for addressing it effectively. Our list of challenges involves *static characteristics* (e.g., absence of orthographic conventions and how it affects data collection), *dynamic processes* (e.g., speed of language change in small languages and how it affects longitudinal collection of big amounts of data), and *interactive relations* between non-dynamic features that are nevertheless subject to potentially rapid change (e.g., absence of standardized assessment tools or estimates for psycholinguistic variables). The identified challenges represent the domains of data collection and handling, participant recruitment, and experimental design. Among other issues, we discuss population limits and degree of power, inter- and intraspeaker variation, absence of metalanguage and its implications for the process of eliciting acceptability judgments, and challenges that arise from absence of local funding, conflicting regulations in relation to privacy issues, and exporting large samples of data across countries. Finally, the ten experimental challenges presented are relevant to languages from a broad typological spectrum, encompassing both spoken and sign, extant and nearly extinct languages.

## Introduction

Advances in the fields of information and communications technology, data-mining and digitalization have led to a rapid increase in available data. In some fields of science, such as neurobiology, “big data is truly, epically big” (Landhuis, 2017, p. 559), thus bringing forward a magnitude of information that can transform entire fields (e.g., the data explosion and subsequent revolution that swept genomics; Tripathi et al., 2016; Landhuis, 2017). In digital humanities, research with big data has already been transformed into a well-structured field of study with specific objects of interest and clearly defined challenges (Kaplan, 2015). In linguistics, and mainly in corpus linguistics, this unprecedented text- and speech-data explosion has also left a profound mark (Hiltunen et al., 2017), that in the words of Mark Liberman, can be thought of as “the modern equivalent of the 17th century invention of the telescope and microscope. We can now observe linguistic patterns in space, time, and cultural context, on a scale three to six orders of magnitude greater than in the past, and simultaneously in much greater detail than before.” (Liberman, 2014).

Big data are available because of the retrieval, storing, analysis, curation, and diffusion of large amounts of information. In a recent European Parliament briefing (Davies, 2016), big data were defined in terms of three ‘V’ words: they have *volume*, they come from a *variety* of sources and in a *variety* of forms, and they exhibit high *velocity*, as they are collected and analyzed in near real-time. However, there is another notion that brings forth a fourth ‘V’ word: *veracity*. This is the notion of “rich data,” which can be defined as “data that’s accurate, precise and subjected to rigorous quality control” (Silver, 2015). As Hiltunen et al. (2017) highlight, rich data, which take more effort to produce, are as crucial in linguistics as they are in other disciplines because they can provide new kinds of evidence about all types of linguistic phenomena.

In this context, the aim of this work is to identify ten experimental challenges that a linguist who sets out to test the empirical basis for some theoretical proposal is likely to encounter, if her aim is to acquire big sets of rich data from small, young, or non-standard languages. Size is not easy to define, as non-standard languages often appear on linguistic continua that have unclear boundaries as to where one language starts and another one stops. As an approximate definition, small linguistic communities would be those that have less than 10,000 first-language speakers, something that is true of more than 55% of the world’s languages according to Ethnologue reports (Simons and Fennig, 2018). Similarly, the definition of a ‘young language’ is not clear in the literature either, but most works characterize as ‘young’ those languages that are only a few decades old (Meir et al., 2010; Schembri, 2010).

In this paper, our emphasis is on the notion of veracity, hence we frequently make a connection between potential discrepancies between one’s experimental results on the basis of a tested sample and the “real-world” conditions that exist at the population level. Put another way, we draw attention to whether a scientific discovery amounts to a true effect (i.e., one that is found in the real world) or whether it is an artifact of data collection and treatment processes. The identified challenges represent the domains of data collection, participant recruitment, and experimental design, leaving aside ethical issues and moral obligations (e.g., aspects of compensation to the tested community) which have been adequately covered in a number of recent works (e.g., Schilling, 2013). Last, apart from identifying and discussing the challenges, we outline recommendations for addressing them effectively, whenever possible.

## Challenge 1: Population Limits, Degree of Power, and Linguistic Landscape

Sample size and adequate power are two factors that determine the validity of research outcomes. Power refers to the long-term probability of rejecting a null hypothesis based on detecting a significant effect in a sample, assuming this effect really exists at the population level (Cohen, 1992). If one’s aim is to obtain a large sample of rich data, power is crucial and a well-powered experiment entails recruiting a large number of participants. A result is more likely to be an accurate reflection of population-level effects in scientific fields that undertake large trials, rather than small ones, for various reasons: (i) an increase in sample size enables a more accurate estimation of effect size (Ioannidis, 2005; Szucs and Ioannidis, 2017), (ii) the effect of individual variation is more likely to significantly impact the global result in small sample experiments, while in large trials this effect will be more neutralized, and the sample will capture a bigger fraction of population-level effects (Braver et al., 2010), and (iii) a small sample size may facilitate obtaining statistically significant—hence, publishable—results that are, however, not doing justice to real-world conditions by means of finding an effect in the selected sample, when there is none at the population (i.e., false positives/Type I errors; Szucs and Ioannidis, 2017).

In research that measures linguistic behavior under experimental conditions, sample size is an important variable that must be decided through taking into consideration the type of language/linguistic community to be tested. Determining what counts as a big vs. small sample size is in itself a complex issue, even more so when one seeks to test behavioral outcomes that represent fluctuating cognitive traits. In psychology, recent estimates place the cut-off for power at *n* > 138 (Bakker, 2015). In cognitive neuroscience, *n* = 50 has been described as a narrowly reasonable sample size, if one conducts whole-brain analyses (Yarkoni, 2009). In linguistics, and specifically in experimental syntax, the elicitation technique in combination with the effect size plays a role in determining sample size. Recent research suggests that when the effect size is small, *n* = 100 does not reach 80% power in experiments that use Likert scales or Magnitude Estimation, while for a medium effect size, 10 participants provide >80% power through the same elicitation techniques (Sprouse and Almeida, 2017).

Assuming, however, that the cut-off for power in experimental syntax is *n* = 10 entails a generalization that comes with a certain risk for various reasons. First, quite often linguists do not present an estimation of effect size, as Sprouse and Almeida (2017) also highlight. Second, sometimes effect size is not being taken into account as a factor at all; a sample size is determined on the basis of what is seen in the literature of the particular area of research in combination to whatever limits are imposed by the funding situation (or the absence of one). If, as is often the case in linguistics, the conducted experiment is an exploratory, hypothesis-generating one rather than a pre-registered, hypothesis-testing one (i.e., that rests on some a priori defined hypotheses about the tested variables and the expected outcomes), a bigger sample size would be needed in order to avoid errors that stem from overestimating the effect size. Third, and more importantly, the origin of this “*n* = 10” must be factored in, because the linguistic landscape matters. To explain this further, the aforementioned sample sizes for experimental syntax were calculated on the basis of speakers who come from big, well-integrated linguistic communities that speak languages with a long history of adaptation, meaning a prolonged period of time during which the language in question is used over a number of generations of first-language speakers (e.g., speakers of English in the United States). However, obtaining *large(r)* samples of participants is a necessity that is more pronounced in *small* and *isolated* languages than in big and well-integrated ones for various reasons.

The first reason is that smaller languages have been associated with greater *complexity* or *diversity*—both terms referring to size inventory in this context—at least in certain aspects of grammar (Lupyan and Dale, 2010; Dahl, 2011; Nettle, 2012). [As a side note: even though in this case we view complexity as size inventory, because this is the meaning that figures in the works we discuss, counting morphemes, syntactic nodes, or other particles that make up an inventory without considering the relations between these and their semantic role does not provide the full picture with respect to complexity (cf. Aboh, 2015)]. Varying degrees of complexity and diversity are the result of a cluster of factors such as (i) population size, (ii) speaker distribution, (iii) degree of interaction among speakers, (iv) adaptation period, during which linguistic features that pose particular difficulties for acquisition are less likely to survive and be passed on to the next cohort, and (v) contact with people that learned the language in question as a second language in adulthood (Trudgill, 2001; Dahl, 2004, 2011; Lupyan and Dale, 2010; Nettle, 2012). Second, contact with adult learners outside the community (i.e., *exoteric communication*) leads to a simplification toward rule-based regularity (Wray and Grace, 2007). This high regularization of irregularities entails a lower degree of violations of the ‘One Meaning–One Form Principle’ which defines as less complex languages that have a transparent, one-to-one correspondence between meaning and form (see Miestamo, 2017 for a recent overview of violations of this principle and their implications for complexity). Taken together these variables suggest that big languages that are long-shaped through adaptation pressures induced by the need to facilitate exoteric communication tend to be *less grammatically specified* (Lupyan and Dale, 2010) and *more ‘regular’* (i.e., having regularized irregularities; Wray and Grace, 2007) compared to small languages spoken in linguistic communities that are oriented toward esoteric communication, that is, communication with members of the community. Under these circumstances, the power cut-off should be set considerably higher in studies that recruit speakers of small and/or more diverse/complex languages. Naturally, population limits and/or speaker distribution in small communities might present a serious challenge for obtaining large and evenly age-distributed samples of informants.

Another related challenge comes from the fact that small linguistic communities often involve language continua and multilingual speakers. To make the comparison with English again, when testing (i) monolingual speakers of a (ii) big language with a long history of adaptation in a (iii) systematically regularized, syntactic phenomenon, the judgments that come from a handful of informants may indeed be representative of population-level conditions, as shown in Sprouse and Almeida (2017) for certain effect sizes. When, however, some of these three variables are not met, the reality that the linguist faces in the field may be quite different. Uniformity gives way to a mosaic of irregularities, interspeaker variation, and psycholinguistically driven language preferences through which a bilingual speaker “projects her identity” (Le Page and Tabouret-Keller, 1985) and which are bound to obfuscate the fieldworker’s quest for specific data. The following description of Skilton’s (2017) fieldwork experience with an endangered language in the dialectally diverse Máíhiki-speaking community in Peru is indicative of how the linguistic landscape affects and constraints sample size and power.

[W]orking with a small number of consultants may conceal both the existence and the structure of variation from the fieldworker. […] As I worked with more consultants, I found that community judgments about speakers’ suitability as consultants rarely agreed with my own. Otilia, labeled by herself and others as ‘speaking wrong,’ turned out to be a fluent, native speaker of both Máíhiki and Spanish (a rare combination) and an excellent consultant. The judgment of her Máíhiki as ‘wrong’ likely stemmed from her use of certain variants stereotyped as indexing the Eastern dialect, which has very low status in this region. […] Likewise, several speakers who others claimed were ‘not from the Algodón,’ such as Trujillo, were in fact born in the Algodón to parents from other settlement areas. […] If I had followed the community’s initial expectations and not worked with people who ‘spoke wrong’ or were ‘not from here,’ I would have captured much less of the extent of variation in the speech community, and I doubt that I would have come to understand any of the social patterning of the variation.

(Skilton, 2017, p. 109–110)

These challenges are the outcome of an interactive relation between population size and linguistic landscape. Although each community is endowed with its own unique characteristics, a general strategy that can maximize power, within the constraints that small and heterogeneous communities impose, could go through the use of age-dispersed populations within a linguistic community. Lastly, it is worth remembering that power is critical in science, and of course linguistics is no exception. As Ioannidis (2005) shows, a large proportion of published results are three things: statistically significant, underpowered, and false. This means that across fields, most published research reports statistically significant results, and yet, these results do not necessarily correspond to a real effect that is *truly attested in real-world conditions*. The issue of false discovery has led to the so-called irreproducibility crisis in science, or put in a simpler way: “If you use *p* = 0.05 to suggest that you have made a discovery, you will be wrong at least 30% of the time. If, as is often the case, experiments are underpowered, you will be wrong most of the time” (Colquhoun, 2014, p. 1).

In the case of theoretical linguistics, statistical calculations might not always apply, but the issue of taking care to not report results that fail to correspond to real-world conditions is still relevant. For instance, if a linguist constructs a theory on the basis of some data that she claims are representative of an understudied language spoken by a small community, but instead they represent only the quirks of her own personal idiolect (possibly due to absence of rigorous testing, for example), this amounts to a false discovery—that is, to finding something that is not truly attested in the real world as described. Schütze (2016, p. 4) remarked on this kind of scenario when he raised the question: “What is to stop linguists from (knowingly or unknowingly) manipulating the introspection process to substantiate their own theories?” In this context, a clear flagging of a study as an exploratory one versus a hypothesis-testing one, together with a clear mention of presence or absence of effect size and sample size calculations, may help the field to reach a better level of transparency.

## Challenge 2: Inter- and Intraspeaker Variation

Whitfield (2008, p. 1370) begins his discussion of striking similarities between the evolutionary paths of languages and species by citing the view of evolutionary biologist Mark Pagel on how “languages are extraordinarily like genomes.”. Genes determine the capacities of organisms, but environmental conditions determine the degree to which these capacities will be developed, also in the case of language (Lewontin, 2000). This view is consistent with what Lupyan and Dale (2010) propose about how language structure is being determined in part by social structure. According to their *Linguistic Niche Hypothesis*, a relationship exists between social structure and linguistic structure such that “the level of morphological specification is a product of languages adapting to the learning constraints and the unique communicative needs of the speaker population” and “the surface complexity of languages arose as an adaptation to the esoteric niche” (p. 7). This hypothesis captures nicely the fact that inter- and intraspeaker variation are dynamic traits that can be found to varying degrees across different linguistic communities as variables *determined by* and in turn *(re)determining* the linguistic landscape. Young, small, and non-standard varieties constitute particularly good landscapes for encountering the challenge of inter- and intraspeaker variation.

To give an example of a small, young, and non-standard language that shows a great degree of variation, let’s consider the case of the nearly extinct sign language used on Providence Island, in the Western Caribbean: Providence Island Sign Language, with 19 deaf signers and known by a speaking population of 2,000 people in 1986, according to Ethnologue reports (Simons and Fennig, 2018). The most plentiful source for this language is William Washabaugh’s book ‘Five Fingers for Survival,’ which presents his fieldwork on Providence Island. Examining the lexicon and syntax of that sign language, Washabaugh (1986) was stuck by two facts: first, the complete absence of signing-correction and signing-consistency; signers do not correct their peers for incorrect signing and there is a great deal of variation in the descriptive signs people use to refer both to everyday objects (e.g., mango, coconut, etc.) and to other people. From the 63 signs he tested with five informants, only two were conventionalized and showed a systematic one-to-one relationship between one form and one meaning, while 70% of the tested signs had three or more variants. The second point of surprise was that the situation was not different on the grammar front. In the author’s words, “context looms so large in the production and interpretation of strings of signs that I could not discern any context independent syntactic rules” (Washabaugh, 1986, p. 58). He further found no evidence for a morphosyntactic disambiguation of word-order through a distinct marking of thematic roles that denote agents, patients, or benefactors, due to absence of fixed or conventionalized word order.

Providence Island Sign Language is by no means unique in this respect. For Nicaraguan Sign Language, Senghas et al. (1997, p. 553) note the existence of verbs that include some use of spatial direction in first generation signers “but not consistently or contrastively; [use of spatial direction] is therefore not yet a morphological device indicating argument structure,” while the second generation makes use of spatial direction on verbs consistently, within and across subjects. Similar observations for systematicity and signing-consistency were made also for Al-Sayyid Bedouin Sign Language: second generation signers are more consistent than their older peers when it comes to marking clause dependencies, thus suggesting that rule-based regularity arises gradually (Sandler et al., 2011). In other words, the emergence of grammar is gradual (see also the discussion on the gradual emergence of grammars in creoles; Mufwene, 2010; Aboh, 2015; Aboh and DeGraff, 2017; McWhorter, 2018), and so is consistency in the use of grammaticalized markers (Sandler et al., 2011).

What these linguistic communities have in common is that they feature a degree of inter- and intraspeaker variation that makes large-scale testing through a variety of techniques (e.g., context free speech, speech prototypically tied to specific genres, elicitation tasks) the only way to capture the real patterns of language use that are attested at the level of the community and at the level of the individual. This variation gives rise to a set of challenges for the linguist. First, the effect size of a phenomenon of interest may be difficult to estimate, as clearly defined, predictable patterns of variation [see (1) for an English example] are either totally absent or highly inconsistent across speakers.

(1a) They were dancing the whole night.
(1b) ^∗^Dancing were they night whole the.

Second, the theoretical prism through which variation is approached in a mainstream linguistic community may not be readily applicable in an experimental setting that involves eliciting and organizing data from speakers of small, young, and non-standard varieties. For instance, Washabaugh (1986) argues that Providence Island data do not square with universalist takes on language variation as being something innately constrained through neatly defined, binary paths, such as the parameters presented within the Principles and Parameters framework (Chomsky, 1986; see Baker, 2003 for graphic illustrations of binary parametric paths, and Boeckx and Leivada, 2013, 2014; Biberauer et al., 2014; D’Alessandro and van Oostendorp, 2017 for recent evaluations of this framework from the perspective of variation). Of course, the focus of generative linguistics is on I-language, not E-language (Chomsky, 1986), but the scientific method requires hypotheses/theories to be tested via rigorous collection of intuitions from multiple informants, hence tapping into multiple I-languages, which, when examined at the level of the community, may be more heterogeneous in communities that feature small, young, and/or non-standard languages, compared to big communities that speak standard languages with a long history of adaptation. In other words, the issue that poses challenges for binary parametric hierarchies boils down to interspeaker and intraspeaker variation or, as Yang (2004) puts it, “adult speakers, at the terminal state of language acquisition, may retain multiple grammars, or more precisely, alternate parameter values; these facts are fundamentally incompatible with the triggering model of acquisition […]. It is often suggested that the *individual variation* is incompatible with the Chomskyan generative program” (pp. 50–51; emphasis added). An important attempt at reconciling intraspeaker variation and I-language has been put forward within the Combinatorial Variability Model (Adger and Smith, 2005; Adger, 2006, 2016), which maintains that the I-grammar generates a *pool of variants*, from which a *choice function* selects the relevant variant according to context.

Of course, inter- and intraspeaker variation are not exclusive to the sign modality, and neither are attested only in languages that are young *and* small *and* non-standard. Research on spoken, non-standard varieties with a long history of adaptation has shown that variation is a recurrent theme in bivarietal/bidialectal/bilectal communities: Cornips (1998, 2006) for Standard Dutch and Heerlen Dutch, Henry (1998, 2005) for Standard English and Belfast English, and Leivada and Grohmann (2017) for Standard Modern Greek and Cypriot Greek, among others, give similar reports on how variable morphosyntactic realizations of the same phenomenon are to be found in the repertoire of dialect speakers.

How should the experimental linguist deal with this variation? Although every linguistic landscape differs and there is no fit-for-all recipe, one way to elicit data that do better justice to the magnitude of population-level variation in communities that feature exceptionally high degrees of variation is to combine elicitation tasks, that target specific linguistic phenomena, with obtaining samples of both naturalistic speech and written texts. With respect to the former, certain genres might offer a better window to variation than others (Skilton, 2017), hence topic variability is advised. Moreover, a way for dealing with variation at the stage of result analysis and interpretation is to search for interaction effects across the various factors that induce variation, through the use of adequate statistical models. For example, in the literature on acceptability judgments, the use of Random Forests and Regression and Classification Trees has been recommended for dealing with unbalanced sets of data (Endresen and Janda, 2016). These statistical models have the potential to extract variable importance among the factors that may drive an effect. They can also deal with variation that is restricted to subsets of the overall set of participants/observations through finding a link between these subsets and specific factors that may influence the final outcome of the experiment.

## Challenge 3: Absence of Corpora of Naturalistic Speech to Complement Experimental Results

Linguists often aim to collect sets of data that target a specific phenomenon in a specific population—for instance, the acceptability of non-verb-second patterns in older adult speakers of Norwegian. At the same time, introspective judgments are *filtered* (and to some extent, possibly distorted) through one’s perceptions about her language or her performance as a speaker. An example of filtering is seen in Skilton’s (2017) presentation of her fieldwork in Peru that was given above: Speakers label themselves or other speakers as “speaking wrong,” even though they are fluent and native in the language tested. This filtering suggests that there may be a discrepancy between a speaker’s introspective judgments about linguistic phenomena and a speaker’s actual productions of these linguistic phenomena. As Cornips and Poletto (2005) put it, one striking result of experimental linguistics is that a speaker may judge a form completely unacceptable in an acceptability judgment task, but still use it productively in her speech.

Indeed, introspective judgments and experimentally elicited production, although at the heart of the empirical base for linguistics and a very useful tool for probing into linguistic behavior, cannot be the only source of evidence for any language, linguistic community, or phenomenon (see Schütze, 2016 for an extensive analysis). Two excellent examples of why this is so—both discussed in detail in Baggio et al. (2012)—come from Labov (1996) and Levelt (1972). In the first study, Labov investigated the use of *anymore* to mean something equivalent to *nowadays*, in the white community of Philadelphia. The results showed a clear mismatch between judgments and actual behavior:

Yet introspective responses to questions about *anymore* are very erratic indeed. In 1973–4, we identified 12 speakers who used positive *anymore* freely though responses to questions of types (1–5) were entirely negative. Jack Greenberg, a 58-year-old builder raised in West Philadelphia, gave introspective reactions that were so convincing that I felt that I had to accept them as valid descriptions of his grammar. Yet 2 weeks later, he was overheard to say to a plumber, “Do you know what’s a lousy show anymore? Johnny Carson.”

(Labov, 1996, p. 85)

As Baggio et al. (2012) note, one might say that this discrepancy between introspective judgments and behavior stems from the fact that naïve informants may not be able to correctly interpret the nature of this task. In other words, it may be the case that variation in the judgments—again, among members of the same community—is restricted to informants who are not able to decipher instructions that ask them to provide introspective judgments about linguistic data. However, Levelt (1972) showed that introspective judgments are far from uniform, even among trained linguists that speak the same language. Let’s consider the well-formedness of (2), which is one of the examples he tested.

(2) The talking about the problem saved her.

(Fraser, 1970, p. 91, with the example marked as ungrammatical)

Upon asking 24 linguists to decide whether (2) would be marked as grammatical or ungrammatical in the original source, Levelt (1972) found that judgments varied: around 1/3 of the consulted linguists gave the judgment ‘ungrammatical,’ thus agreeing with the original source. The rest of the sample (*n* = 17) gave the judgment ‘grammatical.’ This example does not show a mismatch between introspective judgments and production of course (although it is possible that one exists in this case too), but it does reveal how using an acceptability judgment task in a small sample of ten informants may fail to reveal the variation that exists at the population level, even when testing regularized data from big languages with a long history of adaptation: One can easily imagine a scenario under which the ten informants could all come from the aforementioned *n* = 17 sample that gave the judgment ‘grammatical.’ If we also consider that the task of judging a linguistic stimulus is not tied exclusively to the well-formedness of the stimulus itself, but instead involves a range of cognitive processes, in the execution of which, informants might differ considerably (e.g., the ability to mentally construct situations to which the stimulus could be used; Schütze, 2016, p. 111), it is clear that (i) variation should be expected and (ii) running a small-power acceptability judgment task may not reveal the full range of variation.

In this context, it becomes clear that when the aim of the linguist is to elicit big data, results obtained from elicitation techniques should be compared to corpus data of naturalistic speech. As Chomsky (1965, p. 18; emphasis added) puts it, “*the actual data of linguistic performance* will provide much evidence for determining the correctness of hypotheses about underlying linguistic structure, along with introspective reports.” Apart from corpora of naturalistic speech, recent advances in the field of digital humanities have made possible the assimilation of quite large corpora through written texts collected from the internet (e.g., the enTenTen corpus for English with 15 billion words). The challenge is that this range of sources is either very limited or completely unavailable for linguists that work with small, young, or non-standard varieties. Big corpora of rich data are available for big, standard languages. If, however, somebody wants to study specific types of nominalizations in Griko, a variety of Greek spoken in southern Italy, fieldwork is necessary in order to determine the relevant patterns of use in naturalistic speech because the existing corpus resources are restricted to a handful of songs or other texts from similar genres. Doing this fieldwork is definitely a challenge that considerably augments both the time and the financial resources required to complete the research, often making the task impossible to undertake without considerable funding.

## Challenge 4: Absence of Standardized Assessment Tools and Estimates for (Psycho)Linguistic Variables

In the context presented in the discussion of the previous challenge, it is clear that when one runs different types of tasks, many factors come into play; factors for which the experimental linguist that works with big languages might have data, but the one that works with small, young, or non-standard languages probably does not.

To give an example, let’s suppose that we want to administer a self-paced reading task that measures response times to Greek Cypriot people with dementia in order to examine whether reversible passives (e.g., ‘John was seen by Mary’ and ‘Mary was seen by John’) are costlier to process than non-reversible passives (e.g., ‘John was captivated by the music’ and ^∗^‘The music was captivated by John’). We would test the target population in their home variety: Cypriot Greek, which is the non-official, non-standard variety of Greek spoken in Cyprus. If we are to compare linguistic structures in such a task, we will need to make sure that our sentences are matched on a couple of variables, such as syntactic structure, syllable length, lemma frequency, imageability, familiarity, concreteness, and age of acquisition, among others (see e.g., Kambanaros, 2009). Although information about such variables exists for big languages, its absence in Cypriot Greek constitutes a challenge frequently found when one works with small, young, or non-standard languages. Similarly, the absence of standardized assessment tools and norm-referenced tests burden the task of speech pathologists (see Theodorou et al., 2017 on the implications of this absence for diagnostic purposes) and experimental psycholinguists alike.

The paucity of standardized assessments of behavior for determining baseline abilities together with the absence of estimates for psycholinguistic variables such as lemma frequency is a major challenge for the experimental linguist that does not work with big languages. At the same time, it is a challenge that can in part be remedied through the use of assessment tools and psycholinguistic variables from other languages as proxies. For example, in their research with Greek Cypriot children with Specific Language Impairment or word-finding difficulties, Kambanaros and Grohmann (2011) calculated lemma frequencies for object and action names in Cypriot Greek on the basis of the available information for Standard Greek, because at the time no lemma frequency data were available for the tested language. However, one should keep in mind that, although using data from other languages is perhaps the only way to meet this challenge, quite often typologically very proximal languages may show important differences in terms of lemma frequency (e.g., see the comparison between the most frequent verbs per semantic class in Spanish and Catalan in Aparicio et al., 2008).

Adopting a comparative perspective, this challenge, together with the previous one (i.e., absence of corpora of naturalistic speech to complement experimental results), represent non-dynamic features that are nevertheless subject to potentially rapid change. As such, they are challenges possibly easier to overcome time-wise compared to (i) other interactive relations that may fluctuate in time but at a considerably slower rate (e.g., the relation between population size, speaker distribution, and linguistic landscape discussed above) and (ii) more static characteristics such as the difficulty in eliciting introspective judgments from speakers of non-standard varieties, discussed in the next section.

## Challenge 5: Absence of Metalanguage and Difficulty in Eliciting Introspective Judgments in Non-Standard Varieties

Corpora of naturalistic speech offer insights about what is part of a language, but cannot show the actual limits of variation in that language. It is impossible to establish what is *not* acceptable by analyzing corpora of naturalistic speech (Henry, 2005). Native judgments are thus an indispensable tool for the linguist. Obtaining such judgments for a linguistic phenomenon is a process considerably easier when one tests people whose native repertoire includes the relevant metalanguage: for instance, terms such as “acceptable” and “grammatically correct” (the latter being a problematic term, as speakers have intuitions only about what they accept as part of their native repertoire). Put another way, when it comes to collecting judgments on non-standard varieties from dialect speakers, the “real problem […] is in ensuring that speakers know what grammaticality judgements are” (Henry, 2005, p. 1603).

The linguist will of course explain that the nature of the task is to gain insights into the speakers’ native variety and not into what the grammar books define the rules in the standard, but this brings on a second problem: Quite often there is an objective difficulty in conveying this idea and, therefore, in obtaining the relevant data. This difficulty derives from the fact that dialect speakers are aware of the differences that exist between their non-standard repertoires and what is deemed as correct in the standard variety (Henry, 2005; Leivada et al., 2017b). One of the challenges that the linguist thus faces when eliciting introspective judgments from speakers of non-standard varieties is that when these people are asked to use language in order to talk about (their) language, their linguistic behavior may *shift* toward the standard (Labov, 1996). As a matter of fact, being asked to perform in the non-standard variety, in a formal, non-everyday setting such as a sociolinguistic interview or a psycholinguistic experiment, may offer ground for more than a shift. It may lead to a *complete denial* of the ability to perform in the non-standard variety. The following anecdotal piece of evidence from our own research in Cyprus is illuminating. In the process of recruiting participants for our ‘clitics-in-island’ experiment (Grohmann et al., 2012, 2017), we visited various schools to inform them about the purpose of our research. In one of these visits, a native speaker of Cypriot Greek—the school principal, no less—upon hearing that we were interested in testing Greek Cypriot children in their home-variety, first shifted toward the standard while conversing with the first author who is a native speaker of Standard Greek, then denied that Cypriot Greek was heard *at all* in her school, and last, while having just affirmed so, she turned momentarily to some workers to ask them to lay the boxes on the ground more gently, in Cypriot Greek.

This behavior is by no means restricted to a single incident. As Arvaniti (2010) suggests for Cypriot Greek, speakers may often downplay its differences with the standard and reduce it to nothing more than “an accent.” This denial poses an important challenge for the linguist, who is essentially being called to conduct a sociolinguistic interview or a psycholinguistic experiment in something that, in the interviewees’ opinion, does not exist as a system of its own. This is a challenge that one would not face when recruiting graduate students, monolingual speakers of English, through Amazon Mechanical Turk, but is one likely to arise when one works with non-standard varieties.

One way to overcome the ‘absence of metalanguage’ challenge is to replace a question that asks whether a stimulus is acceptable or (grammatically) correct with “Does this sound right to you?” or “Could you say this?” (Henry, 2005). Having groups of native speakers of non-standard varieties discuss acceptability amongst themselves can also help overcome this challenge: where a consensus can be reached, the features under discussion can be assumed to belong to the community of speakers (Henry, 2005). The ‘shift toward the standard’ challenge could be individually assessed as a potential factor of influence through the administration of a background questionnaire that will test the attitudes of a speaker toward the languages that make up her linguistic repertoire (e.g., Papapavlou and Satraki, 2014).

## Challenge 6: Speed of Change in Small Languages

The process of acquiring big data may be a longitudinal one, requiring repeated observations of people from the same or different age groups in different points in time. Longitudinal studies fall in three categories: trend, panel, and cohort studies (Hua and David, 2008). Trend studies test different groups of people from the same population at different points in time. Panel studies test the same, randomly selected, group(s) of people at different points in time. Cohort studies test the same group(s) at different points in time, but each group is selected based on one or more specific characteristics. Crucially, while panel/cohort studies shed light on the process of maturation in the *individual*, trend studies aim to confirm stability in the *population*, with the samples of recruited participants considered “directly comparable” (Sankoff, 2013, p. 262). In other words, in some longitudinal studies, a degree of *uniformity* is presupposed to exist among the members of the linguistic community. When working with small linguistic communities, the acquisition of rich data over a prolonged period of time must take into account factors such as the speed of linguistic change and the degree of uniformity within and across age cohorts.

Let’s analyze these factors separately starting from speed of change. It has been argued that the rate of language change is not cross-linguistically constant; smaller communities have been related to faster change, especially in relation to word loss rate (Nettle, 1999; Bromham et al., 2015). At the same time, the influence of population size on speed of change is not uniform. A recent comparative study confirmed that rates of word loss are significantly greater in smaller languages, but only for the Indo-European family; no effect was found for small languages from other families such as Austronesian or Niger-Congo (Greenhill et al., 2018). This led to the conclusion that either the influence of population size on change rate is not universal or it is weak enough to be mollifiable by other influences (Greenhill et al., 2018). Given that most research proceeds on the basis of a soft heuristic, according to which scientists often select a sample based on what is typically seen in their field of study (Krueger, 2017), it is more challenging to carry out trend studies in small languages, precisely because one cannot follow general heuristics, but rather must take into account factors such as the *potentially* faster rate of change and lower degree of uniformity, in the processes of sample selection and data collection.

In relation to uniformity, some small and/or young languages may be affected by lack of standardization and codification due to their recent emergence, absence of official status, and other sociological factors. Standardization enhances uniformity (Henry, 2005; Leivada et al., 2017b), hence a trend study with monolingual speakers of standard English, French, or Italian may be targeting a more uniform population than a trend study with speakers of Al-Sayyid Bedouin Sign Language, where great differences can be observed both across and within age cohorts (Sandler et al., 2005). Again, the linguistic landscape might differ considerably even across languages and communities that look alike on a number of factors. For instance, Providence Island Sign Language and Al-Sayyid Bedouin Sign Language are two young sign languages that emerged in a short period of time, through the birth of a proportionately large population of deaf individuals in their respective communities in Providence Island and Israel, respectively. Even though they share a number of characteristics, they differ in terms of uniformity. For the former, Washabaugh (1986) reported a *complete absence* of signing-correction among the members of the community. However, correction—together with greater consistency and uniformity—is present in the case of Al-Sayyid Bedouin Sign Language (Meir et al., 2010). The latter is in use among the members of a tight-knit community and this degree of interaction facilitated a rapid growth of consistency and correction, whereas the number of signers on Providence Island was small and their distribution widespread.

The issues identified above present a challenge that boils down not to the conduction of a longitudinal study in small, young, or non-standard languages *per se*, but to the logistics of it in terms of samples and points of observation in time. The soft heuristic for sample selection may work well in big, standardized languages that have a stable rate of change and/or a high degree of uniformity, but small, young, and non-standard languages may require the calculation of additional factors of influence.

## Challenge 7: Absence of Orthographic Conventions

A large part of research in experimental linguistics involves tasks that are implemented in the written modality. Undertaking this research presupposes the existence of some orthographic conventions and a written system. Leaving aside the fact that several of the linguistic communities discussed above lack critical literacy skills (e.g., the Al-Sayyid Bedouin Sign Language and Providence Island Sign Language communities), the absence of a written system and orthographic conventions bring forward an important challenge, even when testing populations that do possess literacy skills in the written modality, however, only having conventions that belong to another language in their repertoire.

For example, in the Greek Cypriot community, people use Standard Greek in the written modality in formal contexts. When writing occurs in the home variety, first it is highly restricted to informal communication, second it takes place through instant messaging applications or social media platforms, and third it reveals a highly inconsistent use of the roman alphabet (Themistocleous, 2010; Ayiomamitou and Yiakoumetti, 2017). As Armosti et al. (2014, p. 23) highlight, the need for codification is evident as there is a range of situations where people “choose to or must write in Cypriot Greek, and hence are inevitably faced with the quandary of how to write in this non-codified variety.”

Under these circumstances, the experimental linguist that works with non-codified varieties faces two problems. The first problem has to do with the presentation of the test stimuli itself. In communities where the presentation of the non-standard variety in a written form looks strange (Henry, 2005 for Belfast English), the solution is the replacement of written tasks with oral questioning. In communities where the non-standard variety can be written even in an inconsistent form, one can resort to the use of dictionaries and thesauri (Leivada et al., 2017a for Cypriot Greek), although such sources often fail to represent the contemporary linguistic reality (Armosti et al., 2014 for Cypriot Greek). The second problem relates to the presentation of other experimental material such as instructions, consent forms, information sheets, etc. This challenge boils down to the aforementioned absence of meta-language in non-standard varieties: If a consent form written in the standard is handed before the experiment, it is likely that this invites a shift toward the standard in speakers’ behavior too, something that may muddle the results, if the aim is to gain insights into the non-standard, home variety. If the consent form is written in the non-standard variety, absence of meta-language will render necessary the incorporation of the relevant terms from the standard, resulting in a strange, even artificially looking mix between the two varieties.

This challenge can be partly addressed through restricting testing to the oral modality (Henry, 2005). However, even in this case, some information may need to be presented in the written modality. For example, the Declaration of Helsinki expresses a clear preference for obtaining a potential subject’s informed consent in writing, further specifying that “if the consent cannot be expressed in writing, the non-written consent must be formally documented and witnessed” (World Medical Association, 2018). Another way to address this challenge is to conduct testing in the written modality too, presenting the test stimuli in whatever convention is largely used in the community, and in parallel assess various sociolinguistic factors that may indicate a shift toward the standard (e.g., gender, education, language attitude toward the home variety, etc.).

## Challenge 8: Absence of Theoretical Descriptions of the Phenomena to be Tested

The abundance of theoretical descriptions of linguistic phenomena in big languages offers a considerable aid in the design of an experimental task through (i) facilitating a clear presentation of the phenomena to be tested together with their predicted exponents or realizations in the target language and (ii) offering a context into which the analysis of the results can be integrated. The field’s overall research focus is often biased away from underexplored language families and isolated linguistic communities (Bakker, 2010), something that suggests that a big number of extant languages lack theoretical descriptions that may provide a valuable reference point across various phases of data collection, analysis, and interpretation.

The bibliographical gap that often exists when conducting research in small, young, and non-standard varieties posits the challenge of not having enough initial information about the way the various phenomena of interest are featured in the target linguistic landscape. This gap considerably increases the exploratory piloting work to be done before any actual experiment, testing, or interview can be run. Another related challenge, and possibly a harder one to overcome, comes not from the total absence of theoretical descriptions of the phenomena/communities to be tested, but from their paucity. To explain this further, one can formulate the *one-source problem* that arises when there is a single source of information or documentation for a so-called “exceptional,” small, isolated linguistic community. This entails that the little available information for such a community is filtered through a single person’s or even team’s perception and interpretation of the facts, which may or may not be verifiable through other sources (i.e., the one-source problem).

One example comes from Pirahã, a small language spoken in Amazonas, Brazil, which has been described as a language that shows certain “inexplicable gaps” such as absence of word structure and recursion (Everett, 2005)—understood in that context as the ability to embed a linguistic unit of one type into another unit of the same type. On the basis of Everett’s work, Pirahã was hailed in the press as a marvelous rarity. Subsequent research from different groups of researchers cast some doubt on the inexplicable gaps and rarities of this language (Nevins et al., 2009). Recent research from Everett himself also shows a perceptible toning down of the exceptionality of the language: “Thus, in summary, we do not find strong evidence of a syntactic relation between sentences and apparent adverbials modifying them, so we cannot determine if recursive embedding is present on the basis of adverbials in the corpus” (Futrell et al., 2016, p. 13). A very positive feature of Futrell et al. (2016) is that they make the corpus on which they base their analysis and claims freely available. This can help overcome the one-source problem, as the same material may be subjected to multiple analyses from different perspectives and people of different theoretical persuasions.

Absence of documentation of the language to be tested entails fieldwork that often requires crossing cultural boundaries. This anthropological challenge, which involves adaptation to the reality encountered in the field of testing, is often neglected by linguists. As Newman (2009, p. 118) puts it, “[f]ield linguists might think that they, unlike the anthropologists, are not investigating anything personal or confidential; but, as uninvited guests in someone else’s society, field linguists often have an impact on many people’s lives of which they may be totally unaware”. Quite often students of linguistics who work on data collection in communities that speak scarcely documented languages struggle with conducting multi-site testing, with assimilating the cultural variability and individual characteristics of each place/community, and/or with getting the right informants in a way that conforms with the societal practices attested in each site. The solution to this challenge is extensive training that familiarizes prospective fieldworkers with the experiences documented through interviewing anthropologists about their experiences in the field (e.g., Pollard, 2009).

## Challenge 9: Absence of Local Funding and Challenges with Crowdsourcing Data

In recent years, much funding of linguistic research has shifted from structural to on-project basis. Many countries established research agendas that focus on some specific research areas to the detriment of others [e.g., the Dutch National Research Agenda (2017) in the Netherlands, DFG Priority Programmes in Germany]. While some, mostly southern European, countries still have structural Ph.D. programs in linguistics, most research in northern European countries takes place on a project basis. The competition to obtain grants is extremely high. In the Netherlands, none of the projects submitted in linguistics by junior PIs were funded in 2017 (NWO Veni results, 2017).

The Dutch research agency NWO, as well as most other European ones, require the applicant to comment on the impact of one’s research. Unfortunately, the documentation and analysis of minority languages is not considered impactful, nor of direct societal relevance, which leaves very little money for research projects with a large part of language documentation.

Given the lack of systematic funds for activities of language documentation, one good way out could be represented by crowdsourcing: involving citizens in the documentation of their own language and collecting the data online. One such attempt was financed for a small-scale project on Abruzzese, an Italo-Romance variety spoken in the upper southern part of Italy. The project, funded by the Royal Netherlands Academy of Science, consisted in the creation of an interactive atlas where speakers could upload conversations or short stories in Abruzzese. On the basis of this pilot, a larger project involving data crowdsourcing was financed within the ERC framework. This project, entitled *Microcontact: Language variation and change from the Italian heritage perspective* includes a very large part of data crowdsourcing, with the aim of identifying speakers who have the right language profile to be then interviewed during fieldwork^1^.

In the PI’s intentions, this crowdsourcing methodology should apply as follows: heritage communities of Italians in North and South America are alerted by local Italian institutions about this initiative. Young speakers record elderly speakers, both heritage speakers (i.e., roughly people born in America to Italian parents) or émigrés (i.e., first generation migrants, who were born in Italy and left when they were rather young to move to America). The aim of the project is to investigate language change in the Italo-Romance varieties spoken by Italian emigrants and their children, in contact with Spanish, Portuguese, French, and English. Heritage speakers have the task to record themselves and their parents, and upload the recordings on the crowdsourcing atlas.

Crowdsourcing is a very good way to implement the Citizen Science enterprise (Irwin, 1995; Bonney et al., 2009; Hand, 2010 and many others), involving speakers in the documentation of their own variety. However, both data crowdsourcing experiments (the small-scale Abruzzese and the large-scale ERC) present many procedural and methodological problems.

The first issue is the (un)reliability of the data. Native speakers listening to the recordings uploaded could identify some cases of “fake data”: some speakers pretended to speak the requested variety while in fact speaking a different one. This is a general problem for linguists, having to do with speakers switching to the standard language spoken in the area or adapting their language to that of the interlocutor (see section “Challenge 5: Absence of Metalanguage and Difficulty in Eliciting Introspective Judgments in Non-standard Varieties”). In fieldwork, this can often be avoided by making sure that the speaker interviewed is actually who they say they are. In a crowdsourcing setting, with no possibility to check that the speaker is who they say they are, and in the absence of a native speaker to check the data, as often happens in large-scale projects targeting a heavily microvariant pool of dialects, the situation becomes trickier. This challenge can be at least partly resolved by following up data crowdsourcing with fieldwork, where possible (but see “Challenge 10: Conflicting Regulations and Privacy Issues”).

The second issue with data crowdsourcing is that it is very hard to persuade people to follow instructions remotely, so for example if one asks for a 5 min conversation they might try to upload 25 min of video. This might be acceptable to a certain extent, but it makes it much more difficult to achieve uniformity of the data format. Furthermore, data protection regulations restrict the kind of data one can put online: some of the data received through crowdsourcing cannot be cleared and put online because they violate too many privacy constraints (see section “Challenge 10: Conflicting Regulations and Privacy Issues”), much to the disappointment of those who did the work of recording and uploading the data.

A related issue is the user-friendliness of the app/website for data crowdsourcing. Many users who are willing to contribute find basic instructions as “record,” “upload,” and so on too difficult. In many cases, elderly people are more interested than the young to document their language, but they are not very much at ease with IT. The only solution for issues such as those is to try many different options and design apps/websites that are as user-friendly as possible. Another solution is to do the crowdsourcing by means of a phone app rather than a website. This reduces the chances that people with little experience with IT will try and upload the data.

## Challenge 10: Conflicting Regulations and Privacy Issues

On May 25, 2018, the General Data Protection Regulation (GDPR) was enforced in the European Union. The GDPR is mainly concerned with data protection and privacy, and requires the enforcement of a number of rules that are not always easy to implement in linguistics.

The basic principle behind privacy is that personal data must be protected in such a way that the person to which these data belong cannot be identified. Personal data are defined as ‘any information relating to an identified or identifiable natural person (‘data subject’); an identifiable natural person is one who can be identified, directly or indirectly, in particular by reference to an identifier such as a name, an identification number, location data, an online identifier or to one or more factors specific to the physical, physiological, genetic, mental, economic, cultural, or social identity of that natural person’ (GDPR, Art4).

For language data, this is a very difficult issue to handle. For instance, it is almost impossible to imagine how the audio data of a research in phonetics or intonation could be handled in such a way that the recorded speaker is not identified. Human voice is unique (Chen, 2016) and recognizable. If voice is recognizable, it is not possible to present it at a conference, or include it in a scientific article where it would be exposed to someone else’s attention. Pseudonymization, which is the usual procedure to handle linguistic data, is certainly not enough to make the speaker unrecognizable.

Personal data must be protected through measures to be defined and implemented by the research institutions. However, at the moment there are no common data protection protocols within the European Union; different institutions implement the GDPR according to what the data protection officer deems important. The result is that data are saved in a different way, thus becoming not comparable, and that researchers are often asked to comply with conflicting instructions.

Concerning crowdsourcing, the GDPR creates many difficulties. We already saw that fieldwork is a very important way to make sure that the data crowdsourced correspond to reality, to ascertain the sociolinguistic profile of the speaker, and double-check the data (see section “Challenge 9: Absence of Local Funding and Challenges with Crowdsourcing Data”). However, the PI and the data protection officer are usually the only people allowed to know the identity of the speakers. If the project starts from the assumption that fieldwork will target those speakers who first uploaded the data via crowdsourcing, this privacy requirement becomes a problem, as the identity of the speakers cannot be revealed to the other members of the research group, in principle. A Ph.D. student cannot know who the speakers are that they should interview in fieldwork and who have first provided the data, without explicit authorization, which comes from state officials, not only from the speakers.

Big data research often resorts to the destruction of the code linking the anonymized file to the speaker. While this has no impact on the soundness of the research, it is in direct conflict with the GDPR requirement to grant the data provider (i.e., the speaker) the possibility to revoke the permission to use their data at any moment. In the absence of an identification key, it is impossible to track the file that the speaker provided.

A further problematic aspect of the GDPR and academic procedures in general is the request of an informed consent. While this is perfectly understandable for sensitive data, it creates several problems in linguistic research. Consent is defined as follows: “Consent should be given by a clear affirmative act establishing a freely given, specific, informed and unambiguous indication of the data subject’s agreement to the processing of personal data relating to him or her, such as by a written statement, including by electronic means, or an oral statement.” (GDPR Recital 32).

Informed consent is based on the assumption that the speaker will understand the purposes of the research, and the aims for which the data will be used. This assumes in other words that speakers are aware of what it means to use linguistic data for scientific inquiry, which is obviously not the case. Furthermore, asking speakers who may be quite old and not very familiar with scientific research, or illiterate, to sign a consent or to declare overtly that they agree on something they don’t understand may cause uneasiness and uncertainty, and in some cases suspicion; furthermore, asking for explicit consent or to sign forms is not always culturally acceptable (Adams et al., 2008 and many others, mainly on medical research).

An additional problem for crowdsourcing is how to ensure that the person providing the consent is the speaker, and not the uploader. One way to solve this problem is to request the uploader to indicate their relation with the speaker. This is, however, not nearly enough: children do not have any legal power on their parents, for instance; if the recording should be performed by, say, school pupils, they would have no legal power to enter the consent for the person interviewed. It is not possible to ascertain that the speaker has actually consented to the use and publication of their data from remote.

As far as international data transfer is concerned, exporting from and to a non-EU country requires two steps: first, the transfer must be legal (according to the GDPR); second:

If the intended data transfer meets the general requirements, one must check in a second step whether transfer to the third country is permitted. One must differentiate between secure and unsecure third countries. Secure third countries are those for which the European Commission has confirmed a suitable level of data protection on the basis of an adequacy decision. In those countries, national laws provide a level of protection for personal data which is comparable to those of EU law. At the time that the General Data Protection Regulation became applicable, the third countries which ensure an adequate level of protection were: Andorra, Argentina, Canada (only commercial organizations), Faroe Islands, Guernsey, Israel, Isle of Man, Jersey, New Zealand, Switzerland, Uruguay, and United States (if the recipient belongs to the Privacy Shield). Data transfer to these countries is expressly permitted.

(GDPR Third Countries).

The GDPR assumes that all countries have the same definition, or even a definition, of data protection. This is obviously not the case. Furthermore, not all countries have protocols in conformity with the GDPR.

One of the countries targeted by the ERC project *Microcontact* is Brazil. Since Brazil is not included among the cleared countries, this means that special agreements must be made to ensure that the data collected in, or exported to, Brazil must be protected in a way which is compatible with the GDPR, and that a data protection officer must ensure that. In the case in which an institution does not have a data protection protocol, in the case in which a country is not concerned with privacy, what is the way to go? Excluding some areas of the world from research collaboration does not appear to be a feasible solution. At the moment, however, there is no option available other than violating the GDPR, or excluding most of the world from research collaboration.

## Outlook

The aim of the work was to present 10 experimental challenges that are likely to be encountered by linguists when embarking on fieldwork in linguistic communities that feature small, young, or non-standard languages. Focusing on the domains of data collection and handling, participant recruitment, and task design, we discussed issues such as degree of power and population limits, the impact of variation, the absence of estimates for (psycho)linguistic variables, the absence of orthographic conventions, the absence/paucity of theoretical descriptions of target linguistic phenomena, the impact of speed of change, the absence of the relevant metalanguage, and the challenges that arise from absence of local funding, crowdsourcing initiatives, and ethics policies. The claim we intend to put forth is that the acquisition of big data (i.e., data that have volume, variety, velocity and, above all, veracity) is relevant for the rigorous testing of all languages, regardless of their size, age, and sociolinguistic status. However, the acquisition of such data may pose more pronounced challenges for the linguist working with small, young, and/or non-standard languages.

As the solutions to these challenges outlined here suggest, the linguistic landscape matters when addressing any of them. Each linguistic community is unique and, as we argue, there is no fit-for-all recipe. Yet, as general guidelines for addressing the challenges identified here, we can highlight the use of big, representative samples, the calculation of effect size, the use of complementary techniques for data collection (e.g., targeted elicitation, interviews, spontaneous speech), the employment of practices that encourage raw data sharing, the clear flagging of a conducted study as an exploratory/hypothesis-generating vs. a hypothesis-testing one, and familiarization with the sociolinguistic and anthropological challenges one may encounter in the field. The list is not exhaustive, which was not our intention; further additions to this line of research are very likely to lead to a better level of transparency in our field.

## Author Contributions

EL and KKG jointly conceived of the paper. EL wrote a first draft, supplemented by RD’A. All authors then equally edited this full draft for submission.

## Conflict of Interest Statement

The authors declare that the research was conducted in the absence of any commercial or financial relationships that could be construed as a potential conflict of interest.

## Footnotes

1 https://microcontact.sites.uu.nl
